# Recent Advances in Electrochemical Sensors for the Detection of Anti-Inflammatory and Antibiotic Drugs: A Comprehensive Review

**DOI:** 10.3390/bios15100676

**Published:** 2025-10-08

**Authors:** Gisele Afonso Bento Mello, Stephen Rathinaraj Benjamin, Fábio de Lima, Rosa F. Dutra

**Affiliations:** 1 Curso de Licenciatura em Química, Instituto de Ciências da Educação (ICED), Universidade Federal do Oeste do Pará (UFOPA), Avenida Marechal Rondon, s/n, Caranazal, Santarém 68040-070, PA, Brazil; gi_abmello@hotmail.com; 2 Laboratory of Behavioral Neuroscience, Drug Research and Development Center (NPDM), Department of Physiology and Pharmacology, Federal University of Ceará (UFC), Coronel Nunes de Melo 1127, Porangabussu, Fortaleza 60430-270, CE, Brazil; 3 Postgraduate Program in Health and Development in the Central-West Region of Brazil, Federal University of Mato Grosso do Sul (UFMS), Campo Grande 79070-900, MS, Brazil; fabio.delima21@gmail.com; 4 Biomedical Engineering Laboratory, Department of Biomedical Engineering, Federal University of Pernambuco, Avenida Professor Moraes Rego, 1235, Recife 50670-901, PE, Brazil; rosa.dutra@ufpe.br

**Keywords:** electrochemical sensor, drugs detection, NSAIDs, antibiotics

## Abstract

Electrochemical sensors have emerged as powerful analytical tools for the detection of anti-inflammatory and antibiotic drugs due to their high sensitivity, rapid response, and cost-effectiveness compared to conventional chromatographic and spectrophotometric methods. This review highlights recent advances in electrode materials, surface modification strategies, and signal amplification approaches for quantifying nonsteroidal anti-inflammatory drugs (NSAIDs) and various antibiotic classes, including sulfonamides, tetracyclines, macrolides, and quinolones. Particular attention is given to nanostructured carbon-based materials, metal nanoparticles, and polymer composites that enhance electron transfer, improve selectivity, and lower limits of detection (LODs). The analytical performance of different electrochemical techniques such as cyclic voltammetry, differential pulse voltammetry, and square-wave voltammetry is critically compared across various drug targets. Trends indicate that hybrid nanomaterial-modified electrodes consistently achieve sub-micromolar detection limits in biological and environmental samples, offering potential for point-of-care diagnostics and environmental monitoring. Current challenges include improving sensor stability, mitigating fouling effects, and ensuring reproducibility in complex matrices. Future research should focus on integrated, miniaturized sensing platforms capable of multiplex detection, paving the way for rapid, portable, and sustainable analytical solutions in pharmaceutical and biomedical applications.

## 1. Introduction

### 1.1. Progress and Challenges Associated with Electrochemical Sensors and Biosensors for Drug Detection

Over the past century, scientific and technological advancements have significantly improved public health, quality of life, and access to medical care. The development and mass production of pharmaceutical compounds, particularly anti-inflammatory and antibiotic drugs, have played a critical role in reducing the global burden of infectious and chronic diseases. Improvements in sanitation, diagnostics, and therapeutic interventions have contributed to decreased infant mortality, increased life expectancy, and enhanced socio-economic conditions worldwide. However, alongside these benefits, the widespread and often unregulated use of pharmaceuticals has led to new challenges, chief among them being the efficient monitoring of drug residues in biological and environmental matrices [1,2,3].

The excessive consumption and improper disposal of pharmaceutical compounds have resulted in their persistent presence in clinical, industrial, and environmental settings. Trace levels of antibiotics and anti-inflammatory drugs are frequently detected in water bodies, wastewater, soil, and food products, often ranging from a few nanograms per liter (ng/L) to micrograms per liter (µg/L), depending on the compound and matrix [4,5]. These residues pose serious concerns, including the promotion of antibiotic-resistant pathogens, the disruption of microbial communities, and potential toxic effects on human and animal health. As a result, the accurate, sensitive, and timely detection of these compounds has become a global priority, driving the demand for reliable and rapid analytical methods [6].

Conventional analytical techniques such as ultraviolet–visible (UV-Vis) spectroscopy, high-performance liquid chromatography (HPLC), gas chromatography (GC), and mass spectrometry (MS) have been widely employed for the detection and quantification of pharmaceutical residues. These methods offer high sensitivity, selectivity, and reproducibility. However, their use is often limited by high instrument costs, laborious sample preparation, long analysis times, and the requirement for sophisticated laboratory infrastructure and trained personnel [7]. These limitations hinder their applicability in decentralized or re-source-limited settings, such as field testing, point-of-care diagnostics, and real-time environmental monitoring.

In response to these challenges, electrochemical sensors and biosensors have emerged as promising alternatives, offering platforms for rapid, cost-effective, and on-site detection of pharmaceutical compounds [8]. These sensors operate by converting the interaction between the target analyte and a chemically or biologically modified electrode surface into a measurable electrical signal. A typical electrochemical sensor comprises a recognition element (e.g., enzymes, antibodies, aptamers, or molecularly imprinted polymers), a transducer (usually a working electrode), and a signal processor or amplifier for quantitative data interpretation.

**Figure 1:**
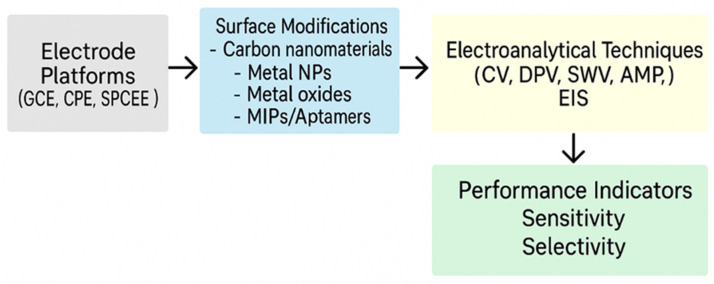
Schematic illustration of the fabrication of an electrochemical sensor showing stepwise integration of nanomaterials and a recognition element on the electrode surface.

The primary advantage of electrochemical sensors lies in their versatility and adaptability. The working electrode can be modified with a wide array of materials including carbon-based nanomaterials (e.g., graphene or carbon nanotubes), metal nanoparticles, conductive polymers, and hybrid composites to enhance conductivity, surface area, and catalytic activity [9,10]. In recent years, the development of novel nanomaterials has significantly advanced the performance of electrochemical sensors for drug detection. Among these, Mxenes, a family of two-dimensional transition metal carbides, nitrides, and carbonitrides, have gained considerable attention due to their high electrical conductivity, large surface area, chemical tunability, and excellent biocompatibility. Their metallic conductivity and hydrophilic surfaces make them particularly suitable for interfacing with biomolecules and enhancing electron transfer in sensor platforms. MXene-based electrochemical sensors have shown remarkable sensitivity and selectivity for pharmaceutical compounds, including antibiotics and NSAIDs, in complex biological and environmental matrices [7,8,9]. These materials can be integrated with polymers, enzymes, or aptamers to create hybrid interfaces that amplify signal output and lower detection limits, positioning MXenes as promising candidates for next-generation point-of-care diagnostics. These modifications significantly improve the sensor’s sensitivity, selectivity, and stability for specific drug targets. Moreover, electrochemical sensors are easily miniaturized, making them well-suited for portable, real-time, and in situ monitoring applications. These modifications significantly improve the sensor’s sensitivity, selectivity, and stability for specific drug targets. These steps are visually illustrated in Figure 1.

The choice of electroanalytical technique is equally critical as it dictates the nature of the analyte–electrode interaction and the signal output. Common techniques include voltammetry, amperometry, potentiometry, and electrochemical impedance spectroscopy (EIS). Voltammetry, which records current as a function of applied potential, is particularly useful for characterizing redox-active drugs. Amperometry, involving current measurements at a fixed potential, is preferred for continuous or real-time analysis. Potentiometry, which detects changes in potential with minimal current flow, is advantageous for monitoring ionic species and pH variations. EIS offers valuable insights into interfacial and molecular interactions, making it particularly suitable for label-free biosensing applications [10].

Each of these techniques has distinct advantages and limitations depending on the analyte, sample matrix, and application. Therefore, their strategic selection and optimization are essential for developing high-performance electrochemical sensors. In the subsequent sections of this review, we explore recent advancements in the design, fabrication, and application of electrochemical sensors and biosensors for the detection of anti-inflammatory and antibiotic drugs. Special emphasis is placed on material innovations, signal amplification strategies, and their real-world applicability in clinical, pharmaceutical, and environmental contexts [11].

### 1.2. Electrochemical Detection Modes in Sensor Applications

The performance of electrochemical sensors is closely linked to the detection mode employed, which governs the sensitivity, selectivity, and analytical resolution of the system. Among the various electroanalytical techniques, cyclic voltammetry (CV), differential pulse voltammetry (DPV), and chronoamperometry (CA) are most frequently used in pharmaceutical and biomedical sensing applications.

Cyclic voltammetry is a versatile method used for probing redox behavior and studying electrochemical mechanisms. By sweeping the potential linearly in both the forward and reverse directions, CV provides valuable information about the oxidation and reduction processes of analytes, including peak potential and reversibility. It is particularly useful in characterizing electrode surfaces and evaluating the electroactive nature of NSAIDs and antibiotics.

Differential pulse voltammetry enhances sensitivity by applying a sequence of potential pulses superimposed on a linear sweep. The resulting current response is measured just before and at the end of each pulse, minimizing background currents and increasing signal resolution. DPV is widely used for trace detection of pharmaceutical compounds due to its high signal-to-noise ratio and low detection limits, often in the nanomolar or even picomolar range.

Chronoamperometry involves the application of a fixed potential over time and measures the resulting current as a function of time. It is particularly suited for real-time or continuous monitoring, where the diffusion-controlled nature of the current response allows for quantification of analyte concentration. CA is especially advantageous in disposable or portable sensor systems, where simplicity and speed are crucial [10].

Each of these techniques offers unique advantages depending on the sensor design and analytical requirements. A concise overview of these techniques and their main analytical parameters is provided in Table 1. Their strategic integration enhances the applicability of electrochemical sensors across pharmaceutical, environmental, and clinical settings.

Carbon-based electrodes, such as glassy carbon electrodes (GCEs), carbon paste electrodes (CPEs), and screen-printed carbon electrodes (SPCEs), have been widely adopted as base platforms for electrochemical sensing due to their excellent conductivity, availability, and compatibility with surface modification. Moreover, several studies have demonstrated that even unmodified versions of these electrodes can effectively detect drugs such as ibuprofen, diclofenac, and aspirin with satisfactory analytical performance [13,23,24].

### 1.3. Anti-Inflammatory Drugs: Description, Common Agents, Therapeutic Uses, and Side Effects

Nonsteroidal anti-inflammatory drugs (NSAIDs) constitute one of the most widely consumed classes of pharmaceutical agents globally, primarily due to their well-documented analgesic, antipyretic, and anti-inflammatory effects. These drugs are essential in the management of pain, inflammation, and fever associated with a range of acute and chronic conditions, including musculoskeletal disorders, osteoarthritis, rheumatoid arthritis, and postoperative recovery. Commonly used NSAIDs include aspirin, diclofenac, ibuprofen, and naproxen, which are frequently available over the counter (OTC) [21,25].

NSAIDs exert their pharmacological effects mainly through the inhibition of cyclooxygenase (COX) enzymes COX-1 and COX-2 which are responsible for the conversion of arachidonic acid into prostaglandins. These prostaglandins are lipid mediators involved in the induction of pain, inflammation, and fever. While the inhibition of COX-2 accounts for the therapeutic effects, COX-1 inhibition is often linked to adverse effects due to its role in maintaining gastrointestinal integrity, renal perfusion, and platelet aggregation. As a result, long-term or unsupervised use of NSAIDs, particularly facilitated by their OTC availability, is associated with several adverse outcomes such as gastrointestinal ulceration, bleeding, nephrotoxicity, hepatotoxicity, and increased cardiovascular risks including myocardial infarction and stroke [26,27].

Beyond their clinical relevance, NSAIDs have emerged as a new class of environmental contaminants. Due to their high water solubility, chemical stability, and partial metabolism in the human body, NSAIDs are often excreted in active forms. These compounds are poorly removed by conventional wastewater treatment plants, resulting in their accumulation in aquatic environments. Residual levels of NSAIDs have been detected in surface waters, rivers, and even drinking water sources, raising concerns over their ecotoxicological impact on aquatic organisms and potential risks to human health through bioaccumulation and chronic exposure [11,28,29,30].

Structurally, NSAIDs are diverse and can be categorized into different chemical classes based on their core functional groups. These include propionic acid derivatives such as ibuprofen and naproxen; acetic acid derivatives like diclofenac; acetylated salicylates exemplified by aspirin; and selective COX-2 inhibitors such as celecoxib. The selective COX-2 inhibitors, while offering reduced gastrointestinal side effects, are less frequently explored in electrochemical sensor research due to their relatively low redox activity com-pared to traditional NSAIDs. Given their widespread use, therapeutic significance, environmental persistence, and health risks, the development of reliable, rapid, and cost-effective detection methods for NSAIDs is imperative. In this context, electrochemical sensors have shown exceptional promise, offering the sensitivity and selectivity needed to detect these compounds in both biological fluids and environmental samples [31]. The subsequent section explores the principles and progress of electrochemical detection methods applied to this important drug class.

#### 1.3.1. Recognition Elements in Electrochemical Sensors

The performance of electrochemical sensors strongly depends on the recognition elements used to confer selectivity toward the target analyte. These components are responsible for molecular recognition and often dictate sensitivity, selectivity, and applicability across different sample types. A variety of recognition strategies have been explored for the detection of NSAIDs and antibiotics: aptamers, molecularly imprinted polymers (MIPs), enzymes, antibodies, and nanomaterials.

Aptamers are short single-stranded DNA or RNA molecules selected for high affinity toward specific targets. Aptamer-based sensors (aptasensors) offer excellent selectivity, reusability, and stability compared with antibodies, and they can be easily modified with nanomaterials to improve electron transfer. Their main limitations are relatively high cost of selection and sometimes reduced performance in complex matrices [32,33]. MIPs are synthetic polymers containing molecular “cavities” complementary to the shape and functionality of the target analyte. They are robust, inexpensive, and stable under harsh conditions, making them attractive alternatives to biological recognition elements, though incomplete template removal and nonspecific binding can reduce selectivity [34,35]. Enzymes provide high specificity and catalytic amplification, often leading to improved sensitivity, but their stability is limited by pH, temperature, and storage conditions [36,37]. Antibody-based sensors (immunosensors) rely on strong antigen–antibody interactions and provide clinically validated specificity, though they are expensive and less stable than synthetic recognition systems [2,38]. Finally, nanomaterials such as metallic nanoparticles, carbon nanostructures, and metal oxides can enhance selectivity indirectly by increasing adsorption affinity, providing catalytic activity, or facilitating electron transfer. Although they do not confer molecular recognition in the classical sense, their physicochemical interactions often mimic recognition functions, particularly in nonbiological sensing applications [39,40].

To better illustrate the comparative advantages of these strategies, Table 2 summarizes the main recognition elements used in electrochemical sensors for anti-inflammatory and antibiotic drugs, highlighting their strengths, limitations, and typical applications.

Overall, the choice of recognition element is strongly application-driven. Aptamers and antibodies provide excellent specificity and are therefore preferred for clinical and biomedical applications, where accurate discrimination in complex biological matrices is critical. However, their higher cost and limited stability can restrict large-scale or long-term use. In contrast, MIPs and nanomaterials offer robustness, low cost, and ease of preparation, which make them attractive for environmental monitoring and pharmaceutical quality control, even though they may suffer from nonspecific interactions. Enzymes remain valuable for signal amplification and selectivity, but their sensitivity to storage and environmental conditions often limits their use in portable devices. Taken together, these comparisons suggest that future sensor development should focus on hybrid platforms that combine the robustness of synthetic recognition elements with the selectivity of biological systems, while also considering cost-effectiveness and scalability for real-world deployment.

#### 1.3.2. Electrochemical Detection of NSAIDs

Electrochemical techniques have rapidly emerged as a powerful and versatile platform for the detection and quantification of NSAIDs in complex matrices such as biological fluids and environmental waters [41,42]. These methods offer distinct advantages over conventional analytical techniques, including high sensitivity, rapid response times, portability, and cost-effectiveness [43]. Importantly, electrochemical sensors are well suited for on-site analysis and point-of-care testing, requiring minimal sample preparation, thereby facilitating real-time monitoring [44].

The electrochemical detection of NSAIDs leverages their intrinsic redox activity, which allows them to participate in electron transfer reactions at the electrode surface [45]. Techniques such as DPV and SWV are widely employed due to their excellent sensitivity and ability to suppress background noise, making them suitable for trace-level analysis. Amperometry is also commonly used for continuous monitoring applications under a constant applied potential [46]. Although less frequently used for direct quantification, electrochemical impedance spectroscopy (EIS) serves as a valuable tool for studying interfacial properties and enhancing selectivity when used in label-free detection schemes.

To improve sensor performance, extensive research has focused on modifying electrode surfaces with advanced materials that promote electron transfer and increase the active surface area [47]. Carbon-based electrodes, such as glass carbon electrodes (GCEs), carbon paste electrodes (CPEs), and SPCEs, have been widely adopted as base platforms. These are often enhanced with nanostructured materials including graphene, rGO, and carbon nanotubes (CNTs), which provide high conductivity, large surface-to-volume ratios, and abundant adsorption sites. Additionally, metal nanoparticles such as gold (AuNPs) [48], silver (AgNPs) [49], platinum (PtNPs) [15], and palladium (PdNPs) [50] are frequently incorporated to increase electrocatalytic activity and improve sensitivity [51].

Metal oxide nanomaterials like zinc oxide (ZnO), titanium dioxide (TiO_2_), and copper oxide (CuO) have also shown potential in boosting redox kinetics and enabling selective interactions with NSAID molecules. Conducting polymers such as polyaniline (PANI), polypyrrole (PPy), and poly(3,4-ethylenedioxythiophene) (PEDOT) contribute to enhanced charge transport and electrochemical stability. Molecularly imprinted polymers (MIPs), engineered to contain selective binding cavities complementary to NSAID structures, offer high specificity and have been increasingly used for recognition elements in electrochemical biosensors [52].

Numerous studies have reported that the integration of these advanced materials enables sensors to achieve nanomolar detection limits and broad linear detection ranges. These platforms have demonstrated high reproducibility and selectivity even in complex matrices, including human serum, urine, and environmental samples like river water and wastewater [53,54]. Furthermore, disposable and low-cost configurations such as SPCEs and DVD-based electrodes enhance practicality and scalability, particularly for environmental surveillance and point-of-care diagnostics [55].

**Figure 2:**
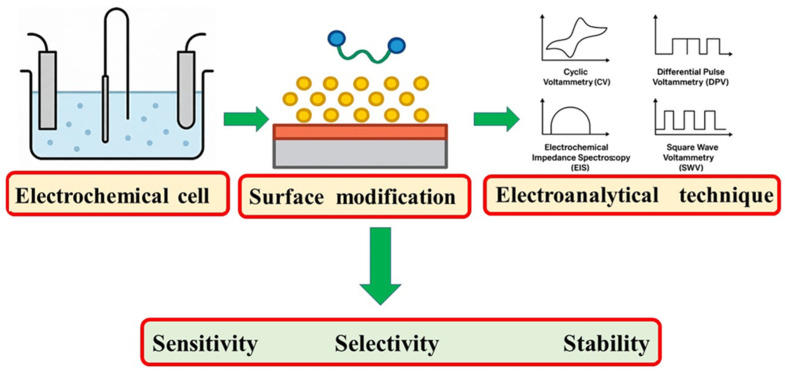
Schematic representation of the design strategy and performance evaluation of electrochemical sensors.

These advancements in electrochemical detection technologies underscore their potential for addressing the analytical challenges posed by NSAID monitoring. In the sections that follow, specific NSAIDs such as naproxen, ibuprofen, diclofenac, and aspirin will be discussed in greater detail, focusing on their redox behavior, electrode modification strategies, and analytical performance in electrochemical sensing systems. Figure 2 illustrates the general design strategy for electrochemical sensors, including electrode platforms, surface modification techniques, electroanalytical methodologies, and their impact on key performance indicators such as sensitivity, selectivity, and stability. Representative electrochemical sensor configurations, detection limits, and materials used for anti-inflammatory drugs are summarized in Table 3.

#### 1.3.3. Electrochemical Detection of Naproxen

Naproxen, a propionic acid-derived NSAID, is electrochemically active and amenable to detection via voltametric methods. The detection mechanism involves an initial irreversible electron transfer-driven decarboxylation, followed by oxidation of the resulting intermediate, ultimately leading to an alcohol derivative. These reactions occur at relatively high anodic potentials, making the design of efficient electrode surfaces crucial to lowering overpotentials, enhancing current response, and reducing interference.

In recent years, there has been significant interest in integrating carbon-based nanomaterials (e.g., graphene, graphene oxide, reduced graphene oxide, and carbon nanotubes), metal and metal oxide nanostructures, cyclodextrin-based molecular inclusion systems, and hybrid composite interfaces. These modifications aim to simultaneously optimize analytical sensitivity, selectivity, and robustness for naproxen detection, particularly in complex sample matrices such as pharmaceuticals, biological fluids, and environmental water.

Among carbon-based materials, graphene oxide (GO) has consistently shown superior electrochemical response towards naproxen. The modification was carried out via electrochemical deposition of graphene oxide nanosheets, which increased the electroactive surface area and enhanced electron transfer between the analyte and electrode surface. This modification improved both the sensitivity and selectivity of the assay. This performance is largely attributed to the abundant oxygen-containing functional groups (e.g., hydroxyl, epoxide, and carboxyl), which enhance the adsorption and catalytic oxidation of naproxen. Qian et al. [11] conducted a comparative study using GO and doped GO derivatives, including fluorine-doped GO (F-GO), boron-doped partially reduced GO (B-rGO), and nitrogen-doped partially reduced GO (N-rGO), along with thermally reduced graphene oxide (TrGO). Using CV and DPV, they demonstrated that pristine GO exhibited the highest peak current and most favorable oxidation behavior for naproxen. This result was strongly correlated with the higher oxygen content on the GO surface, facilitating enhanced catalytic oxidation. Moreover, the GO-based electrodes showed a broad linear dynamic range, high sensitivity, and excellent selectivity even in the presence of potentially interfering species commonly found in biological systems. Notably, the sensors were capable of analyzing commercially available naproxen tablets without sample pretreatment, underscoring the robustness and real-world applicability of the GO-modified platforms.

Metal nanoparticles (e.g., silver nanoparticles) combined with β-cyclodextrin (β-CD) and carbon-based matrices have also demonstrated exceptional performance. These systems leverage the host–guest inclusion capacity of β-CD to selectively preconcentrate naproxen at the electrode surface, while the metallic component facilitates electron transfer and improves redox kinetics. For instance, Tarahomi et al. [63] developed a sensor based on a AgNPs@GO-β-CD nanocomposite immobilized on a low-cost gold DVD electrode, which exhibited enhanced electrocatalytic response and increased stability. The synergistic combination of GO’s high surface area, β-CD’s molecular recognition, and AgNPs’ conductivity resulted in a significantly lower LOD and better signal-to-noise ratios compared to nonfunctionalized electrodes.

Various metal oxides such as ZnO, Al_2_O_3_, ZnFe_2_O_4_, and Cu_3_TeO_6_ have been explored in both standalone and hybrid configurations for their redox-active properties and ability to lower charge transfer resistance (Rct). CeO_2_ nanofibers were synthesized via a hydrothermal process and incorporated onto the electrode through drop-casting, providing oxygen vacancy sites that facilitated catalytic activity and improved analyte adsorption. Their incorporation into GCEs or carbon paste electrodes (CPEs) enhances the active surface area and facilitates fast charge transfer kinetics. For example, ZnO-modified GCEs have demonstrated enhanced peak currents and lower oxidation potentials for naproxen, attributed to the oxide’s high isoelectric point, defect density, and electron mobility. Similarly, Al_2_O_3_/GCE sensors have shown significant signal enhancement by promoting electrostatic interactions with the carboxylate group of naproxen, which leads to improved accumulation and detection efficiency [64].

The integration of quantum dots (QDs) such as graphene quantum dots (GQDs), CdTe QDs, and nitrogen-doped GQDs (N-GQDs) into electrochemical sensors has opened avenues for achieving ultrasensitive detection in complex biological and environmental samples. These nanomaterials offer unique features, including size-tunable band gaps, high surface area, and abundant sites for bioreceptor conjugation. In particular, QD–AuNP hybrid systems have been used as platforms for aptamer immobilization, creating biosensors with high specificity, ultralow LODs (in the femtomolar range), and strong resistance to nonspecific adsorption. Such biosensors are particularly useful for selective naproxen detection in serum, urine, and wastewater, where matrix interferences are a concern. Electrochemical sensors developed for naproxen have shown impressive analytical performance, with LODs ranging from low nanomolar to sub-micromolar levels. These sensors typically exhibit broad linear dynamic ranges and rapid response times, making them suitable for diverse analytical applications. Notably, many platforms have been successfully applied to the detection of naproxen in real-world matrices such as commercial pharmaceutical formulations (e.g., tablets and capsules), biological fluids (e.g., urine and serum), and environmental samples (e.g., surface water and wastewater). Disposable formats, including screen-printed electrodes (SPEs) and gold DVD-derived electrodes, offer additional advantages by enabling cost-effective, scalable, and field-deployable sensing solutions.

Among the various sensing strategies, GO based sensors and hybrid composites incorporating GO, metal nanoparticles, and β-cyclodextrin (β-CD) stand out for their high sensitivity, selectivity, and broad applicability. However, several challenges persist. Biological and environmental matrices often contain interfering substances that can cause signal suppression or enhancement, requiring the development of matrix-tolerant or self-cleaning electrode surfaces. Furthermore, electrode fouling due to the accumulation of naproxen oxidation products can impair sensor performance over time, necessitating the integration of antifouling strategies or surface regeneration techniques. Another critical issue is the lack of standardized protocols and consistent reporting of analytical parameters such as LOD, linear range, stability, and inter-laboratory reproducibility, which limits the ability to compare and benchmark sensor performance across studies.

To address these limitations and further improve sensor robustness, future research should prioritize the development of multiplexed sensing systems that combine molecularly imprinted polymers (MIPs) or aptamers with GO–metal nanocomposites. The integration of antifouling coatings or surface modifications will also be essential to preserve sensor functionality in complex sample matrices. Electrode surface modifications play a crucial role in enhancing the analytical performance of electrochemical sensors. Common modification techniques include drop-casting, where nanomaterials or polymers are dispersed in solution and deposited onto the electrode surface; electrodeposition, which allows for precise control of material thickness and morphology; and self-assembled monolayers (SAMs), which form highly ordered functional layers. These methods facilitate the incorporation of nanomaterials such as carbon nanotubes, graphene derivatives, metal nanoparticles (e.g., AuNPs and AgNPs), and metal oxides (e.g., Fe_3_O_4_ and ZnO) that significantly improve electron transfer, surface area, and biorecognition capabilities. The advantages of such modifications include increased sensitivity, enhanced selectivity, improved detection limits, and faster response times. However, potential drawbacks include variability in reproducibility, decreased long-term stability, potential desorption of modifiers, and added fabrication complexity or cost. Therefore, selecting an appropriate modification strategy requires balancing performance gains with fabrication feasibility and application-specific demands. Moreover, establishing standardized analytical validation protocols will facilitate the cross-comparison of results and support the broader implementation of electrochemical sensors for naproxen in pharmaceutical, biomedical, and environmental applications.

## 2. Electrochemical Detection of Ibuprofen

Ibuprofen (IBP), a widely used NSAID, is structurally classified under propionic acid derivatives, which include analogs like naproxen and ketoprofen. Its electrochemical behavior is primarily governed by the presence of an aromatic ring system and a carboxylic acid group, which allows for characteristic oxidative decarboxylation pathways. The electrooxidation of ibuprofen typically initiates with the loss of the carboxyl group, followed by electron transfer processes involving the phenylpropionic acid backbone [43]. These mechanisms make it feasible to detect ibuprofen electrochemically, particularly using modified electrodes that improve sensitivity, selectivity, and lower the detection limits.

Carbon-based nanomaterials such as multiwalled carbon nanotubes (MWCNTs), graphene, GO, and rGO have frequently been utilized due to their high electrical conductivity, large electroactive surface area, and excellent chemical stability. Incorporation of MWCNTs into composite matrices enables the immobilization of biomolecules such as enzymes (e.g., cytochrome P450), which can mediate electron transfer and mimic metabolic pathways for ibuprofen oxidation. For instance, a biosensor comprising cytochrome P450 immobilized on MWCNTs was reported to monitor ibuprofen in real time, useful in pharmacokinetic studies and in vitro drug metabolism assessments [11]. Metal-decorated carbon nanomaterials, such as Ag-zeolite/MWCNT composites, further enhance the electrochemical response by synergistically increasing the electroactive area and providing catalytic centers for ibuprofen oxidation [65]. These hybrid electrodes have reported detection limits as low as 10–30 nM, with improved current response and reduced overpotentials.

BDDEs are a class of synthetic diamond materials doped with boron atoms to achieve conductivity while maintaining extreme chemical inertness. BDDEs provide a wide potential window, low background current, and excellent resistance to fouling, making them highly suitable for analyzing complex pharmaceutical matrices. Electrochemical detection of ibuprofen using BDDEs has achieved acceptable recoveries (>95%) in commercial tablet formulations with minimal sample pretreatment [66]. In biological samples, BDDEs still require deproteinization or other cleanup procedures due to biofouling and matrix interferences.

Transition metal oxides such as CuO, ZnO, and particularly complex oxides like Cu_3_TeO_6_ or NiFe_2_O_4_ have shown promising electrocatalytic behavior toward ibuprofen oxidation. These oxides are typically immobilized on conductive carbon substrates to leverage their redox-active surface and defect-induced catalytic sites. The incorporation of metal oxides not only improves adsorption and electron transfer rates but also enhances stability under repetitive scan cycles. For example, a Cu_3_TeO_6_/CNF-modified electrode exhibited a wide linear range (0.1–100 µM) and a low LOD (~6 nM), demonstrating its suitability for pharmaceutical quality control and trace detection in environmental samples [28].

Recent developments have explored the integration of nanostructures like CdTe quantum dots (QDs), nitrogen-doped graphene quantum dots (N-GQDs), and gold nanoparticles into aptamer-based sensing platforms for ibuprofen detection. These platforms utilize aptamers synthetic oligonucleotides that exhibit high affinity and selectivity toward ibuprofen as molecular recognition elements. When combined with QDs or AuNPs, these constructs offer enhanced electron transfer, higher aptamer surface loading, and reduced interfacial resistance [67]. Such platforms have reported ultralow LODs (<5 nM) and excellent selectivity against common interferents like dopamine, uric acid, and ascorbic acid [16].

The move toward practical applications has led to the development of SPCEs, DVD-derived gold electrodes, and low-cost microfluidic formats. These platforms are attractive for environmental monitoring and point-of-use pharmaceutical diagnostics due to their portability, ease of mass production, and minimal need for external instrumentation. Recent SPCE platforms modified with rGO/AuNP composites have demonstrated good reproducibility (RSD < 5%), stable performance across multiple runs, and successful detection in spiked human plasma samples. The hybrid electrode containing AuNPs and reduced graphene oxide demonstrated a synergistic effect—AuNPs provided catalytic hotspots, while graphene offered high conductivity, resulting in a lower detection limit and broader linear range compared to sensors with only one material [29].

Across platforms, ibuprofen LODs have reached the low nanomolar to sub-micromolar range (typically 1–100 nM), depending on the electrode modification strategy. High selectivity is commonly achieved using molecularly imprinted polymers (MIPs), aptamers, or β-cyclodextrin (β-CD) systems, which recognize the target molecule via size or structural complementarity. However, recurring challenges include electrode fouling (especially in biological fluids), interference from electroactive species, and the lack of device standardization. Few studies have validated results using certified reference materials or conducted inter-laboratory comparisons, which are necessary for clinical or regulatory adoption [68].

### 2.1. Electrochemical Detection of Naproxen and Ibuprofen: Mechanistic Insights and Advancements in Sensor Platforms

Naproxen and ibuprofen are widely used NSAIDs belonging to the 2-arylpropionic acid class. These compounds possess carboxylic acid functional groups, making them electroactive and detectable via voltametric and amperometric techniques. The electrochemical oxidation of both naproxen and ibuprofen is primarily governed by an irreversible decarboxylation mechanism, followed by further oxidation of the resulting intermediate. This sequential mechanism results in the formation of an alcohol derivative as the terminal product. The electrochemical behavior is significantly influenced by electrode surface properties, solution pH, and scan rate, necessitating sensor platforms with high electrocatalytic activity and surface functionality for reliable detection [69].

Initial detection strategies utilized carbon paste electrodes (CPEs) and screen-printed electrodes (SPEs), often modified with multiwalled carbon nanotubes to enhance the electron transfer rate and active surface area. The incorporation of MWCNTs markedly improved sensor sensitivity and facilitated the incorporation of biological recognition elements, such as cytochrome P450 (CYP). Carrara et al. [70] developed a MWCNT-based platform for naproxen detection, leveraging the bioelectrocatalytic activity of CYP isoforms. These sensors demonstrated the capability to monitor therapeutic concentrations of naproxen over a 16 h period in human serum, with excellent stability and a low background current.

Further advancements employed metal oxide nanoparticles, notably ZnO and AgZ composites, in hybrid structures with CNTs. Tashkhourian et al. [57] demonstrated that ZnO-MWCNT-modified GCEs exhibited a significantly lower oxidation potential and increased peak current for naproxen. The enhanced electrochemical behavior was attributed to the high isoelectric point, oxygen vacancies, and defect-rich structure of ZnO, which improved electron transport kinetics. Similarly, Motoc et al. [71] developed an AgZ-MWCNT composite sensor for ibuprofen detection. The Ag–zeolite combination conferred excellent electrocatalytic activity and signal stability, allowing for sensitive detection in complex matrices. These hybrid electrodes outperformed unmodified GCEs and even conventional MWCNT sensors in terms of peak current intensity and LOD.

Quantum dots (QDs), particularly CdTe QDs and nitrogen-doped graphene quantum dots (N-GQDs), have been explored for their unique optical and electrochemical properties. Shahdostfard and Roushani [68,72] employed CdTe QDs in aptasensor formats for ibuprofen, taking advantage of their high surface area, photostability, and favorable energy levels for electron transfer. These platforms, integrated with AuNPs and immobilized ibuprofen-specific aptamers, enabled highly selective detection at femtomolar levels. The use of N-GQDs/AuNP nanohybrids further improved aptamer immobilization efficiency and electrochemical responsiveness, establishing a versatile platform for label-free biosensing. The resulting sensors showed excellent reproducibility, low LODs, and negligible interference from structurally similar compounds, making them highly suitable for biomedical diagnostics.

Boron-doped diamond electrodes (BDDEs) offer significant advantages for NSAID detection due to their wide potential window, chemical inertness, and low capacitive background. Švorc et al. [13] reported the successful voltametric detection of ibuprofen in pharmaceutical formulations and spiked human urine using unmodified BDDEs, highlighting their robustness and minimal fouling in real sample matrices. Graphene oxide and its doped variants (N-GO, S-GO, and B-GO) have also been evaluated for naproxen detection. Qian et al. [12] observed that pristine GO exhibited the highest electrochemical signal, likely due to its high density of oxygen-containing functional groups which enhance adsorption and electron transfer. Doping with heteroatoms altered the electronic structure but slightly diminished the current response, indicating the critical role of oxygen functionalities in naproxen electrooxidation.

Mutharani et al. [73] developed a sensor based on Cu_3_TeO_6_ nanostructures deposited on GCE, which exhibited superior electrocatalytic performance for naproxen detection. The unique morphology of Cu_3_TeO_6_ provided a high surface area, and its mixed oxide composition facilitated rapid electron transport. The reported detection limit was significantly lower than that of unmodified electrodes, and the sensor demonstrated good selectivity and stability over repeated measurements. Lima et al. [28] fabricated a sensor using Al2O3-modified GCEs, which significantly enhanced the anodic peak current of both ibuprofen and naproxen. The improved response was attributed to the adsorption-mediated preconcentration of the drugs on the Al_2_O_3_ surface and its interaction with the carboxylate group of the analytes.

Recent developments have focused on nanohybrid composites that combine metal nanoparticles, conducting polymers, and carbon nanomaterials to exploit synergistic effects. Electrode surface modifications play a crucial role in enhancing the analytical performance of electrochemical sensors. Common modification techniques include drop-casting, where nanomaterials or polymers are dispersed in solution and deposited onto the electrode surface; electrodeposition, which allows for precise control of material thickness and morphology; and self-assembled monolayers (SAMs), which form highly ordered functional layers. These methods facilitate the incorporation of nanomaterials such as carbon nanotubes, graphene derivatives, metal nanoparticles (e.g., AuNPs and AgNPs), and metal oxides (e.g., Fe_3_O_4_ and ZnO) that significantly improve electron transfer, surface area, and biorecognition capabilities. The advantages of such modifications include increased sensitivity, enhanced selectivity, improved detection limits, and faster response times. However, potential drawbacks include variability in reproducibility, decreased long-term stability, potential desorption of modifiers, and added fabrication complexity or cost. Therefore, selecting an appropriate modification strategy requires balancing performance gains with fabrication feasibility and application-specific demands.

For instance, Au@f-CNT/GO/GCE sensors demonstrated by Asadpour-Zeynali et al. [30] exhibited superior conductivity and analyte adsorption, leading to ultralow LODs and high reproducibility. Similarly, CNF/Au/PANI/CPE sensors developed by Afzali et al. [74] used carbon nanofibers (CNFs), AuNPs, and polyaniline (PANI) to achieve enhanced electron transfer, surface area, and anti-fouling properties. These systems are particularly useful for detecting naproxen in complex biological samples. A comparison of analytical performance parameters for naproxen- and ibuprofen-based electrochemical sensors is presented in Table 4.

β-cyclodextrin (β-CD), known for forming host–guest complexes with hydrophobic molecules, has been employed to increase sensor selectivity. Lenik and Nieszporek et al. [75] developed a β-CD-modified MWCNT membrane-based potentiometric sensor for ibuprofen, showing stable potential response and low detection limits due to enhanced molecular recognition. Tarahomi et al. [63] fabricated a β-CD-functionalized AgNP@GO composite sensor on a gold DVD platform, enabling the efficient preconcentration of naproxen through host–guest interactions. This sensor showed excellent reproducibility, long-term stability, and resistance to matrix interference, making it promising for real-world applications.

**Table 4:** Comparative Performance of Electrochemical Sensors for Naproxen and Ibuprofen.

Target Drug	Electrode/Modification	Technique	LOD (µM)	Sample Matrix	Reference
Naproxen	GO/GCE (various dopings)	DPV	1.900	Pharmaceutical tablets	[12]
Naproxen	CdS-modified GCE	SWV	0.00143	Pharmaceuticals	[76]
Ibuprofen	Ag–zeolite/–MWCNT composite	CV / DPV	0.00018	Water samples, pharma	[77]
Naproxen and Ibuprofen	Al_2_O_3_-modified GCE	SWV	0.012	Biological, pharmaceuticals	[28]
DCF, Naproxen, Ibuprofen	CNF/GR-CNT paste electrode	SWV	6.08 × 10^−6^–2.86 × 10^−3^	Pharmaceuticals	[18]
Ibuprofen	Bare BDDE	CV / DPV	0.410	Pharmaceuticals, urine	[13]
Ibuprofen	Cu_3_TeO_6_/GCE	CV / DPV	0.017	Pharmaceuticals	[73]
Ibuprofen	TGA/CdTe QD aptasensor	SWV	3.33 × 10^−7^	Pharmaceutical samples	[72]
Ibuprofen	Apt/AuNPs@N-GQDs nanocomposite	SWV	0.03333	Pharmaceuticals	[72]
Naproxen	CPE/FeNi_3_/CuS/BiOCl	Chronoamperometry	0.060	Pharmaceutical samples	[78]
Naproxen	Ni-Fe LDH/Au electrode	CV / DPV	0.001	Pharmaceuticals	[79]

### 2.2. Electrochemical Detection of Aspirin

Aspirin (acetylsalicylic acid, ASA) is an acetylated derivative of salicylic acid, structurally composed of a benzene ring and a carboxylic acid group, bearing chemical similarities to other NSAIDs such as naproxen and ibuprofen. The electrochemical detection of aspirin typically involves a two-electron, two-proton transfer process. Initially, aspirin undergoes hydrolysis to form salicylic acid. This is followed by the oxidation of salicylic acid to form a radical intermediate, which subsequently undergoes further oxidation to produce either 2,3-dihydroxybenzoic acid (2,3-DHB) or 2,5-dihydroxybenzoic acid (2,5-DHB), with the latter being more commonly reported as the principal electrochemical product [23].

The pioneering electrochemical studies on aspirin were conducted using edge plane pyrolytic graphite electrodes (EPPGEs). Goyal et al. [67] investigated the electrochemical behavior of aspirin using SWV on EPPGE and reported significant advantages, such as a broad potential window, low background current, excellent long-term stability, reproducibility, and heightened sensitivity and selectivity. Compared to basal plane pyrolytic graphite electrodes, EPPGEs showed a lower oxidation peak potential and higher current response. These characteristics enabled simultaneous detection of aspirin and caffeine in beverages and determination of unmetabolized aspirin in urine samples.

To enhance the electrochemical response, Kruanetr et al. [80] modified EPPGEs with graphene nanosheets. The graphene-enhanced electrode exhibited improved electrical conductivity, resulting in higher oxidation peak currents and superior sensitivity. These modifications facilitated direct analysis of aspirin in pharmaceutical formulations and oral fluid samples without pretreatment, further establishing the potential of graphene modified EPPGEs as a sensitive platform for real sample detection.

Cuprous oxide (CuO), a p-type semiconductor known for its low charge transfer resistance and favorable redox properties, has been incorporated into GCE for aspirin detection. Sivakumar et al. evaluated various CuO-modified GCEs (CuO-1, CuO-2, and CuO-3) using cyclic voltammetry and amperometry. Among them, CuO-2/GCE demonstrated superior electrochemical activity, offering high selectivity, reproducibility, and sensitivity. The method proved effective for quantifying aspirin in pharmaceutical tablets [69].

Similarly, Muthusankar et al. [81] developed a hybrid electrode composed of N-doped carbon quantum dots (N-CQDs) and Cu_2_O on a GCE. The NCQD/Cu_2_O composite displayed enhanced electron transfer, low resistance, and high conductivity, attributes credited to the N-CQD’s surface structure and synergistic interaction with the Cu_2_O (111) crystalline plane. This sensor exhibited a low detection limit, broad linear range, and high stability, demonstrating excellent potential for ASA detection in real samples.

Molecular imprinting offers an approach for enhancing selectivity through the formation of specific recognition sites. Wang et al. [82] developed an aspirin-specific MIP-based sensor by electropolymerizing p-aminothiophenol (p-ATP) on a Au electrode doped with AuNPs, forming the p-ATP–AuNPs/Au electrode. Cyclic voltammetry results indicated excellent sensitivity, selectivity, and reproducibility, attributed to the increased number of effective recognition sites and the high conductivity conferred by AuNPs.

Polyaniline (PANI), a conductive polymer with excellent electron transfer kinetics, has been integrated with metal oxides to fabricate advanced aspirin sensors. Puangjan et al. [82] fabricated a nanocomposite electrode consisting of PANI, manganese dioxide (MnO_2_), and antimony trioxide (Sb_2_O_3_) on fluorine-doped tin oxide (FTO). This PANI/MnO_2_-Sb_2_O_3_/FTO electrode exhibited a significantly higher anodic peak current, attributed to the synergistic effect of the nanocomposite constituents. The enhanced surface area and conductivity led to improved sensitivity, wide linear range, low detection limit, and high selectivity, even in complex matrices such as urine.

Polymeric materials have been widely utilized in the development of electrochemical sensors due to their excellent film-forming ability, biocompatibility, chemical stability, and capacity to uniformly disperse nanostructured materials. When combined with carbon-based nanostructures or metal nanoparticles, these polymers enhance the electrocatalytic performance of the modified electrodes, thereby improving the sensitivity and selectivity of aspirin detection. Among the most commonly employed polymers are Nafion, chitosan, and poly(resorcinol).

Nafion, a perfluorinated sulfonic acid polymer known for its high ionic conductivity and mechanical stability, has been used in combination with graphene nanofibers to fabricate composite-modified glassy carbon electrodes (GR-NF/GCEs). This modification led to an increased electroactive surface area and significantly enhanced oxidation peak currents for aspirin, facilitating its sensitive and reliable quantification [83]. In another approach, Nafion was used to modify SPCEs in conjunction with electrochemically reduced graphene oxide (ER-GO). The Nafion/ER-GO-modified SPCEs demonstrated rapid electron transfer and robust sensor response, enabling accurate detection of acetylsalicylic acid in complex sample matrices such as pharmaceutical preparations and biological fluids [84].

Chitosan, a natural biopolymer derived from chitin, has also been employed for its excellent biocompatibility, film-forming nature, and strong ability to adsorb analyte molecules. When combined with AuNPs on the surface of SPCEs (SPCE/(CS + AuNPs)), the resulting nanocomposite electrode exhibited enhanced adsorption of aspirin and improved electron transfer kinetics. This synergistic effect contributed to increased sensitivity and signal stability, making the sensor suitable for point-of-care or field-based applications [85].

Another notable polymer is poly(resorcinol), which has been electropolymerized onto the surface of GCE to form a stable and conductive film. The poly(resorcinol)-modified GCE showed significantly enhanced oxidation currents for aspirin, attributed to its increased surface area, better analyte diffusion, and strong π-π interactions between the polymer and aspirin molecules [86]. The incorporation of such conducting polymers provides a tailored microenvironment that facilitates efficient electron exchange between the analyte and electrode surface. Collectively, these polymer-based modifications not only improve sensor stability and reproducibility but also offer a versatile platform for the fabrication of high-performance electrochemical sensors tailored for aspirin detection.

### 2.3. Electrochemical Detection of Diclofenac: Mechanistic and Analytical Perspectives

Diclofenac (DCF), a widely used NSAID, has garnered significant attention due to its environmental persistence and biomedical relevance. From an electrochemical standpoint, DCF exhibits irreversible oxidation behavior, making its detection and mechanistic elucidation critical for applications ranging from pharmaceutical quality control to environmental monitoring. The electrochemical oxidation of diclofenac proceeds via two primary mechanistic pathways.

In the first pathway, oxidation initiates at the nitrogen center of the diclofenac molecule. This involves a one-electron transfer that generates a nitrogen-centered radical cation, which subsequently undergoes molecular cleavage. The resulting degradation products include 2,6-dichloroaniline a potentially toxic aromatic amine and 2-(2-hydroxyprop-2-enyl)phenol. This pathway generally occurs at more anodic potentials and is characterized by significant structural fragmentation [87].

The second mechanism is more complex and involves a sequential multistep process. Initially, a single-electron transfer at the nitrogen forms a radical cation, which loses a proton to generate a carbon-centered radical. A second electron transfer leads to a carbocation intermediate that, in the presence of water, undergoes nucleophilic attack and aromatic rearrangement [88]. This process ultimately yields 5-hydroxy diclofenac [89]. This mechanism is particularly relevant under aqueous or biological conditions and bears resemblance to enzymatic hydroxylation pathways observed in drug metabolism.

A wide array of modified electrodes has been developed for the detection of diclofenac, offering improved sensitivity, selectivity, and reliability. Polymer/carbon composite electrodes, for example, demonstrate outstanding performance due to their synergistic properties. Chitosan–copper–MWCNT nanocomposite coatings on GCEs exhibit remarkable electrocatalytic activity: chitosan enhances biocompatibility, copper nanoparticles boost conductivity, and MWCNTs provide a high surface area and facilitate electron transfer [90,91]. These sensors offer long-term stability (up to nine weeks), high sensitivity, and detection limits comparable to high-performance liquid chromatography (HPLC), making them suitable for pharmaceutical and environmental analysis.

Molecularly imprinted polymer (MIP)-based carbon paste electrodes represent another innovative approach. A polymeric matrix composed of polyaniline and triphenylamine, combined with rGO, creates specific binding sites for diclofenac molecules. These MIP-based sensors offer exceptional selectivity and sensitivity, with analytical performance closely aligned with HPLC methods for pharmaceutical tablets [91]. Further advancements involve nanocellulose and functionalized carbon nanotube composites. Nanocellulose enhances hydrophilicity and mechanical integrity, while functionalized MWCNTs improve electron transport and reduce aggregation. The resulting electrodes offer high peak stability and reproducibility, facilitating accurate detection in complex biological samples such as urine and blood [16]. Integration of conducting polymers such as poly(3,4-ethylenedioxythiophene) (PEDOT) with titanium dioxide nanoparticles and ionic liquids (e.g., [BMIM]Cl) into carbon paste electrodes has also enhanced signal resolution, electron transfer, and operational durability [92]. These modifications improve the viability of electrochemical detection systems as practical alternatives to chromatographic methods, conductive polymers such as polyaniline and polypyrrole were employed to improve film stability and provide additional binding sites for analyte molecules, yielding recoveries of 90–94% in serum.

Metal nanoparticle-based sensors have emerged as highly efficient platforms for diclofenac detection. Gold nanoparticles combined with MWCNTs on a GCE significantly boost electrocatalytic oxidation, offering broad linear detection ranges (0.8–250 µmol L^−1^) and low detection limits (as low as 0.1 µmol L^−1^), with robust operational stability up to five weeks [64]. Bimetallic configurations, such as gold–platinum nanoparticles deposited on functionalized MWCNTs, further enhance redox activity and conductivity, enabling detection in real-world matrices, including pharmaceutical formulations and biological fluids [93]. Platinum nanoflowers on reduced graphene oxide (PtNF–rGO) coatings applied to screen-printed carbon electrodes represent an advanced example. These nanostructures exhibit extremely low charge transfer resistance and high oxidation currents, achieving detection limits in the nanomolar range (down to 9.4 nmol L^−1^), enabling ultratrace analysis [15].

Screen-printed electrodes (SPEs) modified with metal oxides are attractive for disposable and field-deployable detection. Zinc oxide-modified SPEs treated with low-temperature plasma show enhanced surface hydrophilicity and conductivity, resulting in detection limits around 0.012 µmol L^−1^ [94]. Cerium dioxide nanoparticle-modified SPEs, post heat treatment under vacuum, display high catalytic activity and reasonable detection limits (LOD: 1.1 µmol L^−1^) with a linear range of 2–200 µmol L^−1^. While limited in terms of shelf life, these sensors are cost-effective and suitable for point-of-care and environmental applications.

Cyclic voltammetry is predominantly used for mechanistic studies, whereas SWV and DPV are preferred for quantitative detection due to higher sensitivity and lower capacitive background currents. Reported linear detection ranges span from low nanomolar concentrations to those in the several hundred micromolar range, with detection limits ranging from 0.012 µmol L^−1^ to 1.1 µmol L^−1^, depending on the electrode material and configuration. These systems demonstrate strong operational stability, excellent selectivity in the presence of common interferents, and reliable performance in diverse matrices such as blood, urine, and pharmaceutical formulations [24]. 

Comparative assessments indicate that advanced nanostructured electrodes can match or exceed traditional chromatographic techniques, particularly in portability, cost-effectiveness, speed, and minimal sample preparation. Incorporating nanomaterials such as graphene, carbon nanotubes, and metal nanoparticles significantly improves signal amplification and electron transfer kinetics. The inclusion of ionic liquids and molecularly imprinted polymers enhances target specificity. Collectively, these innovations enable the development of robust, reproducible, and sensitive electrochemical platforms for routine diclofenac monitoring. Given diclofenac’s widespread use and environmental impact, electrochemical detection methods provide promising solutions for real-time monitoring in clinical diagnostics and environmental surveillance. The increasing emphasis on miniaturized and disposable sensors particularly in screen-printed configurations further underscores their potential for integration into portable, point-of-care analytical systems.

### 2.4. Simultaneous Electrochemical Detection of Naproprex, Ibuprophen, and Diclofenac

Several materials have been investigated for the electrochemical detection of anti-inflammatory drugs, and depending on the molecular structure of the drug and the analytical conditions, the composition and structure of the electrode directly influence the electrochemical performance. Moreover, as environmental samples often contain multiple pharmaceutical residues due to improper disposal by industries and households the ability to simultaneously detect and quantify individual drugs is crucial for public health.

In this context, Motoc et al. [77] recently developed and evaluated carbon-based electrodes for the simultaneous detection of diclofenac, naproxen, and ibuprofen in real surface water samples collected from the Bega River in Timisoara, Romania. The study utilized fullerene–carbon nanofiber paste (FULL/CNF) and graphene–carbon nanotube (GR/CNT) composite electrodes, along with DPV, chronoamperometry and multiple-pulse amperometry (MPA) for detection. To fabricate the GR/CNT electrode, graphene (GR) and CNTs were blended with paraffin oil to form a conductive paste. Similarly, the FULL/CNF electrode was made by mixing fullerene (FULL) and carbon nanofibers (CNFs) with paraffin oil. The ratio of carbon nanostructures was adjusted to achieve suitable paste consistency, which in turn affected the surface morphology. FULL, characterized by a low specific surface area, contrasts with CNT and CNF, which have moderately higher surface areas, and graphene, which offers the highest. The incorporation of more GR into the GR/CNT electrode increased its porosity and surface area, thereby enhancing its electrocatalytic activity compared to the FULL/CNF electrode.

Cyclic voltammetry was used to study the electrochemical behavior of the individual drugs and their simultaneous detection. On the GR/CNT surface, the oxidation peaks of the analytes were separated by less than 0.2 V, whereas on the FULL/CNF electrode, greater peak separations were observed: 0.2 V between diclofenac and naproxen, and 0.55 V between naproxen and ibuprofen. This suggests that the FULL/CNF electrode provides a better resolution for simultaneous detection using CV. This may be attributed to structural interactions such as π–π stacking or analyte entrapment within the carbon matrix that enhance electron transfer rates. Despite similar LODs on both surfaces, the authors recommended the FULL/CNF electrode for simultaneous detection of the three drugs based on CV performance. Further DPV analyses revealed that the oxidation of diclofenac interfered with the signals of naproxen and ibuprofen, and naproxen also influenced the oxidation of ibuprofen, indicating a hierarchy of electrochemical responses. Finally, when applied to real water samples from the Bega River, both the FULL/CNF and GR/CNT electrodes exhibited distinct oxidation potentials, good selectivity, precision, and reproducibility, confirming their suitability as electrochemical sensors for simultaneous voltametric analysis of naproxen, ibuprofen, and diclofenac, with a particular emphasis on the effectiveness of the FULL/CNF configuration.

## 3. Antibiotics Drugs (Antibacterial Drugs)

### 3.1. Description, Main Popularly Antibiotics Drugs, General Proposes, and Side Effects

Antibiotics are essential medicines widely used across all age groups to treat bacterial infections ranging from minor ailments, such as sore throats and earaches in childhood, to more serious conditions in adults, including urinary tract infections, bronchitis, conjunctivitis, otitis, pharyngitis, skin infections, and diarrhea. Among the most commonly used antibacterial agents are penicillin V, penicillin G, and amoxicillin (Penicillins); doxycycline (Tetracyclines); cephalexin (Cephalosporins); ciprofloxacin and levofloxacin (Quinolones); clindamycin (Lincosamides); azithromycin (Macrolides); trimethoprim (Sulfonamides); vancomycin (Glycopeptides); streptomycin (Aminoglycosides); ertapenem and imipenem (Carbapenems); and metronidazole (Nitroimidazoles) [95]. Given the wide spectrum of antibiotics and their diverse applications, it is crucial to recognize that these drugs are pathogen-specific, and indiscriminate use without medical guidance not only compromises therapeutic efficacy but also causes adverse effects ranging from nausea, vomiting, and diarrhea to severe toxicities, including allergies, myelotoxicity, carcinogenicity, reproductive disorders, hepatic and renal insufficiency, as well as immunopathological complications such as anaphylaxis and autoimmune diseases [96]. Furthermore, inappropriate antibiotic use drives the emergence of resistant bacterial strains, diminishing drug effectiveness or necessitating higher dosages, thereby amplifying the risk of adverse effects. This growing resistance represents a global public health challenge, motivating extensive research into antibiotic monitoring and detection in clinical, veterinary, and environmental samples, particularly through electrochemical methods, with particular focus on widely consumed drugs such as amoxicillin, cephalexin, and azithromycin [97]. Recent electrochemical sensors reported for major antibiotic drugs are summarized in Table 5.

### 3.2. Amoxicillin (Pinicillin Drugs): Description, Action Mechanism, and Electrochemical Detection

Amoxicillin, a widely used antibiotic, is a semisynthetic derivative of penicillin belonging to the β-lactam class of antibiotics. The discovery of penicillin dates back to 1928 when Alexander Fleming observed inhibition of bacterial growth around a mold contaminant in his Staphylococcus aureus culture. The mold, later identified as Penicillium, produced a substance that was named penicillin and marked the beginning of the antibiotic era. The core structure of penicillin and its derivatives is the β-lactam ring, which consists of three carbon atoms and one nitrogen atom [98]. Modifications at this ring’s side chains give rise to different penicillin derivatives with varying antibacterial spectra and pharmacological properties. Amoxicillin, in particular, is chemically known as D-α-amino-p-hydroxybenzyl penicillin trihydrate and is used extensively in the treatment of infections of the ear, nose, throat, skin, and respiratory tract in both humans and animals.

Amoxicillin acts by inhibiting the bacterial cell wall synthesis. It targets and binds to penicillin-binding proteins (PBPs), which are transpeptidase enzymes responsible for the final cross-linking of the peptidoglycan layer in bacterial cell walls. Amoxicillin mimics the D-alanyl-D-alanine moiety of the peptidoglycan precursor and irreversibly binds to the active site of PBPs. This binding prevents the proper cross-linking of the peptidoglycan strands, leading to a weakened cell wall [99]. The compromised cell wall cannot withstand osmotic pressure, resulting in bacterial lysis and cell death. This mechanism makes amoxicillin particularly effective against Gram-positive bacteria that have a thick peptidoglycan layer.

Electrochemical detection of amoxicillin has been widely studied due to the need for sensitive and cost-effective methods for pharmaceutical, environmental, and clinical analysis. However, amoxicillin does not produce a well-defined oxidation peak on unmodified carbon-based electrodes such as glassy carbon, graphite, carbon nanotubes, or even metal electrodes like gold and platinum. Therefore, various surface modifications are required to enhance its electrochemical response. For instance, Santos and colleagues modified a GCE using polyglutamic acid crosslinked with glutaraldehyde (PGA/GLU). This modification improved peak currents in cyclic voltammetry due to electrostatic interactions between the amino group of amoxicillin and the carboxylic groups of PGA. Optimal performance was achieved with a ratio of 87.5% PGA to 12.5% glutaraldehyde, showing good sensitivity for detecting amoxicillin in human urine using SWV, without any prior sample treatment [100].

Further advancement was made by Chen et al. [72] who combined PGA with three-dimensional porous graphene (3D-GE), forming a PGA/3D-GE/GCE composite. This electrode exhibited superior charge transfer capability and enhanced conductivity due to the synergistic effect between the components. The modified sensor demonstrated excellent electrocatalytic activity and performed reliably in real human urine samples, showing high selectivity and reproducibility. Another approach by Deroco et al. [22] involved the use of carbon black (CB) incorporated into a dihexadecylphosphate (DHP) film, forming a CB–DHP/GCE. The porous and nonuniform surface of this sensor significantly improved electron transfer and enabled simultaneous detection of amoxicillin and nimesulide in complex matrices without interference.

Valenga et al. [101] proposed a Nafion and reduced graphene oxide-modified GCE to overcome the limitations of unmodified electrodes. The rGO provided a high surface area and oxygenated functional groups that facilitated electron transfer, while Nafion contributed to the selectivity of the sensor. The modified electrode showed well-defined redox peaks, with a detection limit of 0.36 mmol L^−1^ and a quantification limit of 1.2 mmol L^−1^. It demonstrated good selectivity even in the presence of benzylpenicillin and humic acids, although higher concentrations of humic acids somewhat interfered with sensor performance. This system was effectively used to detect amoxicillin in river water samples.

In another study, Debalke et al. [102] employed a metal–organic complex, diaquabis(1,10-phenanthroline) copper (II) chloride (A2P2CuC), to modify a GCE. The resulting sensor showed a two-fold increase in electroactive surface area and improved electron transfer properties. It provided low onset potential, high current response, good sensitivity, and excellent stability for detecting amoxicillin in pharmaceutical tablets and blood serum. Similarly, Brahman et al. modified a CPE with polyaniline film in a micellar medium, significantly increasing oxidation current and shifting peak potential. The high adsorption capability of the polyaniline film allowed effective accumulation of amoxicillin, making the sensor stable, precise, and suitable for pharmacokinetic studies.

Another innovative design by Ojani et al. [103] involved a Ni–curcumin complex-modified carbon paste electrode (Ni/CR/CPE), prepared via electropolymerization in an alkaline medium. This sensor exhibited a high electron transfer rate and remarkable sensitivity toward amoxicillin. It remained stable for four weeks and showed strong selectivity, enabling its application in real samples. These advancements collectively illustrate that effective electrochemical detection of amoxicillin requires specific surface modifications using nanomaterials, polymers, and metal complexes. These tailored interfaces enhance sensitivity, selectivity, and stability, enabling amoxicillin detection in complex matrices such as biological fluids, environmental samples, and pharmaceuticals.

**Table 5:** Electrochemical Sensors for Antibiotic Drugs.

Drug	Electrode Material	Method	LOD	Reference
Amoxicillin	GCE/quantum dots/PEDOT:PSS	SWV	50 nM	[104]
	Paper based electrode	DPV	8 μM	[105]
	SPE/cobalt-doped titanium dioxide	SWV	5.8 μM	[17]
Ciprofloxacin	CPE/choline chloride	SWV	0.036 nM	[106]
Gentamicin	graphene oxide–gadolinium oxide/SPCE	CV	0.424 pM	[107]
Tetracycline	CPE/Magnetic nanoparticles–MIP	SWV	0.15 μM	[108]
Doxycycline	SPCE/Ni.Gr	SWV	0.0096 μM	[109]
Erythromycin	GCE /Arg-MIP	SWV	2.01 nM	[110]
Rifampicin	GCE/Fe_3_O_4_NPs@MWCNT	DPV	0.64 μM	[111]
Clarithromycin	GCE/molecularly imprinted polyarylene phthalide	DPV	0.053 μM	[112]
Azithromycin	SPCE/4-aminobenzoic acid	DPV	0.08 μM	[113]
Kanamycin	GCE/Co, Mo@CNFs	DPV	2.56 pmol·L^−1^	[114]
Chloramphenicol	GCE/poly (eriochrome black T)	DPV	11 nmol L^−1^	[115]
Levofloxacin	GCE/PEDOT/Chitosan	DPV	0.4 nM	[116]
Streptomycin	GCE/molecularly imprinted composite	DPV	0.25 pM	[117]
Metronidazole	GCE/f-Co@rGO	DPV	0.015 nM	[118]
Cephalexin	GCE/MIP	DPV	3.2 nM	[119]

## 4. Ciprofloxacin

Ciprofloxacin (CIP) is a synthetic second-generation fluoroquinolone antibiotic with potent bactericidal properties, acting through the inhibition of bacterial DNA gyrase and topoisomerase IV, thereby disrupting DNA replication. It is primarily excreted unchanged in urine, achieving high concentrations in urinary tract infections, and to a lesser extent via feces. While particularly active against Gram-negative bacteria such as *Escherichia coli*, *Pseudomonas aeruginosa*, and *Klebsiella pneumoniae*, its Gram-positive activity is more limited. Clinically, CIP is administered orally, intravenously, or topically to treat urinary tract infections, respiratory tract infections, prostatitis, bone infections, and anthrax exposure. Beyond therapeutic applications, CIP has gained attention as an analytical target due to its frequent occurrence in biological, pharmaceutical, and environmental matrices, necessitating sensitive, selective, and cost-effective detection methods.

Recent electrochemical sensing strategies for CIP have demonstrated significant progress in sensitivity, selectivity, and practicality. For example, GCEs modified with hierarchical electrospun carbon nanofibers and NiCo nanoparticles (eCNF/CNT/NiCo-GCE) achieved a LOD of 6.0 µmol L^−1^ and excellent stability for four weeks in complex samples such as urine, plasma, and pharmaceutical formulations [120]. Copper-based metal–organic frameworks (MOFs) have been employed for highly selective CIP detection [121], while β-cyclodextrin-modified silver nanoparticles enabled ultrasensitive, one-pot detection strategies [122]. Similarly, β-cyclodextrin-functionalized carbon nanotubes enhanced electrode performance through host–guest interactions with CIP molecules [123]. Nafi-on/multiwalled carbon nanotube composite films provided selective detection via electrostatic interactions [124], and reduced graphene oxide sensors allowed rapid quantification in pharmaceutical products and milk [125]. Metal oxide nanomaterials such as V_2_O_5_ nanoparticles [126] and noble metal-based electrodes, including gold nanoparticle-modified screen-printed carbon electrodes [127], further improved analytical performance. Other innovations include polymer-modified GCEs without nanomaterials, demonstrating that functional polymer films alone can achieve high sensitivity [128], and cost-effective graphene sheets derived from pencil lead [86]. Moreover, hybrid electrodes combining activated carbon, gold nanoparticles, and supramolecular solvents [129] have achieved excel-lent recovery rates in complex matrices. Collectively, these advances highlight the diverse material strategies available for CIP sensing, ranging from metal–organic frameworks to nanocarbon composites, enabling detection in both clinical diagnostics and environmental monitoring.

### 4.1. Cephalexin (a Cephalosporin Drug): Description, Mechanism of Action, and Electrochemical Detection

Cephalosporins are a class of β-lactam antibiotics, with cephalexin being one of the most widely used representatives. It is commonly prescribed for the treatment of respiratory tract infections such as pneumonia and pharyngitis, as well as bone and joint infections, β-hemolytic streptococcal throat infections, and otitis media. Due to its broad-spectrum activity against both Gram-positive and Gram-negative bacteria and favorable safety profile, cephalexin is also widely used in dermatological applications [119,130,131]. Additionally, cephalexin is recommended for patients with penicillin allergies or those undergoing dental or upper respiratory tract procedures (e.g., nose, mouth, throat, or larynx), particularly individuals with pre-existing cardiac conditions, to prevent infective endocarditis [132].

The mechanism of action of cephalexin is analogous to that of other β-lactam antibiotics, such as amoxicillin. It targets bacterial cell wall synthesis by inhibiting the transpeptidation step of peptidoglycan formation, a critical component of the bacterial cell wall [89,90]. Electrochemical detection of cephalexin has garnered attention due to its analytical significance in pharmaceutical and biomedical fields. As with amoxicillin, initial studies showed limited electrochemical response using unmodified commercial electrodes [133]. Therefore, numerous studies have focused on modifying electrode surfaces to improve the sensitivity and selectivity of electrochemical cephalexin detection.

One such study by Feier et al. [132] examined the electrochemical oxidation of the cephalosporin nucleus at high potentials using DPV with a boron-doped diamond electrode (BDDE). This method was applied to pharmaceutical formulations (commercial capsules), biological fluids (human urine), and environmental samples (river water). The study demonstrated a simple and sensitive detection approach based on the anodic oxidation peak of the cephalosporin structure. All cephalosporins investigated exhibited irreversible anodic peaks, with ceftazidime and cephalexin showing the lowest and highest oxidation potentials, respectively. Notably, cephalexin produced the most pronounced electrochemical signal. The method also showed good selectivity and a low detection limit, supporting its application for real sample analysis.

Subsequently, the same research group developed a molecularly imprinted polymer (MIP)-based electrochemical sensor for cephalexin detection in river water and pharmaceutical samples [119]. The MIP was synthesized by electropolymerizing indole-3-acetic acid (I3AA) onto glassy carbon and BDDE surfaces. I3AA was selected due to its ability to form noncovalent interactions such as hydrogen bonding, electrostatic forces, and π-π stacking with cephalexin, thus enhancing detection selectivity. Electrochemical impedance spectroscopy revealed that the MIP increased the electrode’s porosity, thereby facilitating electron transfer and catalytic activity. The BDDE-MIP platform exhibited higher sensitivity due to a greater number of imprinted cavities, while the GCE-MIP showed better reproducibility, attributed to the more homogeneous GCE surface. However, both electrodes were considered nonreusable due to stability limitations. Importantly, the MIPs demonstrated good selectivity toward cephalexin over other cephalosporins, which is attributed to structural differences in side chains preventing nonspecific binding. These sensors successfully detected cephalexin in pharmaceutical and river water samples with high sensitivity and low detection limits, with the GCE-MIP being preferred due to superior surface uniformity.

In a more recent study, Kassa et al. [131] explored the simultaneous electrochemical detection of cephalexin and cefadroxil using a glassy carbon electrode modified with poly(resorcinol) (poly(reso)/GCE). This system was applied to commercial tablet formulations and biological fluids (human serum and urine). Cyclic voltammetry and electrochemical impedance spectroscopy showed that polymer modification increased the electrode’s surface area and conductivity, enhancing oxidation currents and reducing onset potentials indicating improved electrocatalytic activity. SWV demonstrated that poly(reso)/GCE enabled the sensitive and selective detection of both analytes, even at low concentrations and in the presence of interferents. Furthermore, the modified electrode exhibited good stability, retaining performance after 20 days, which supports its application in routine electrochemical detection of cephalosporins.

### 4.2. Azithromycin (Macrolide Drugs): Description, Action Mechanism and Electrochemical Detection

Azithromycin is a broad-spectrum macrolide antibiotic belonging to the azalide subclass, characterized by a large 15-membered lactone ring. It is extensively prescribed for the treatment and prevention of respiratory tract infections such as pharyngitis, pneumonia, chronic bronchitis, bronchopneumonia, sinusitis, otitis media, and various skin infections. Beyond respiratory ailments, azithromycin is widely employed in managing typhoid fever, toxoplasmosis, sexually transmitted infections, and infections associated with Helicobacter pylori and AIDS-related opportunistic pathogens. In recent years, it has also gained attention for its use, often in combination with other drugs, in the treatment protocols for patients infected with SARS-CoV-2 (COVID-19) [134,135,136,137,138].

The primary antibacterial mechanism of azithromycin involves the inhibition of protein synthesis through binding to the 50S ribosomal subunit, thereby suppressing bacterial growth. In addition, azithromycin can penetrate bacterial extracellular vesicles an important defensive secretory system and exhibits immunomodulatory properties by reducing pro-inflammatory cytokine production, inhibiting neutrophil infiltration, and modulating macrophage polarization. These multifaceted actions contribute to its efficacy against a broad range of microorganisms [86]. Regarding electrochemical detection, most reported studies focus on modified electrode platforms to enhance sensitivity, selectivity, and reproducibility. Such modifications often involve nanomaterials, molecularly imprinted polymers (MIPs), or composite films to achieve low detection limits and reliable quantification, as will be discussed in subsequent sections.

Electrochemical strategies leveraging molecularly imprinted polymers (MIPs) and advanced nanomaterials have delivered remarkably enhanced performance in detecting azithromycin (AZY). For instance, Rebelo and colleagues developed a SPCE modified by electropolymerizing 4-aminobenzoic acid in the presence of AZY as a template. This MIP-based sensor demonstrated a linear detection range between 0.5 and 10 µM and achieved a LOD as low as 0.08 µM, with successful application to tap water and river samples [113]. Complementing this, Pogăcean et al. reported a graphene-modified glassy carbon electrode (EGr/GC) fabricated via electrochemical exfoliation of graphite rods. This sensor exhibited significantly improved sensitivity 0.68 A/M, three times higher than that of bare glassy carbon and an impressively low LOD of 3.03 × 10^−9^ M over a wide linear range (10^−8^ to 10^−5^ M), especially effective in complex matrices due to its high selectivity and stability [139].

Building on these advances, more recent innovations employ novel electrode modifications that combine structural and functional improvements. Jiwanti et al. introduced a screen-printed carbon electrode modified with boron-doped diamond nanoparticles (BDDNP) and rGO, achieving a reliable linear response to AZM in the 30–100 μM range with an LOD of 1.6 μM. Notably, the sensor showed outstanding analytical performance when applied to hospital wastewater, delivering a recovery of 93.3% and a low relative standard deviation (%RSD) of 2.41%, underlining its robustness in environmental monitoring [140]. Another promising platform employs vanadium dioxide (VO_2_) thin-film electrodes synthesized via hydrothermal treatment, serving as an efficient sensing material for azithromycin (AZI) detection. Electrochemical characterization across varying scan rates, pH values (5–8), and AZI concentrations revealed a large active surface area and abundant active sites, attributed to the high porosity of the VO_2_ film. The sensor exhibited pH sensitivities of 61.5 mV pH^−1^ (CV) and 60.0 mV pH^−1^ (SWV), indicating robust stability and responsiveness. Under AZI concentrations ranging from 1.0 to 80 μmol L^−1^, limits of detection and quantification were 0.02 and 0.06 μmol L^−1^ (CV) and 0.025 and 0.084 μmol L^−1^ (SWV), respectively. Combining excellent sensitivity with operational simplicity, this VO_2_-based sensor demonstrates strong potential for applications in pharmaceutical quality control and aquatic environmental monitoring [141].

Ensafi et al. [134] developed an electrochemical sensor by modifying a GCE with a MgCr_2_O_4_ spinel–MWCNT nanocomposite, producing the MgCr_2_O_4_-MWCNT/GCE configuration. The combination of hydrophobic and electrostatic interactions between the spinel and the drug facilitated azithromycin adsorption and enhanced electron transfer, resulting in a lower oxidation potential and higher oxidation peak current compared with bare GCE- or MWCNT-modified surfaces. The electrode demonstrated good stability over 10 successive measurements, reproducibility, and selectivity for AZM determination, with a low detection limit. Performance comparison with conventional techniques confirmed its potential as a selective, simple, and accurate platform for the voltametric detection of AZM in pharmaceutical formulations, plasma, and urine.

In the same year, Zhang et al. [135] reported a “necklace-like” graphene oxide–multiwalled carbon nanotube nanohybrid electrode for AZM detection. When compared with GC, GO/GC, and MWCNT/GC electrodes, the GO–MWCNT/GC electrode exhibited the highest electro-oxidation activity, attributed to the synergistic effects of GO and MWCNTs. The well-distributed GO sheets on MWCNTs provided strong adsorption capacity for AZM via hydrogen bonding, hydrophobic forces, and electrostatic interactions, enhancing both adsorption and electron transfer. In real sample analysis (capsules and human urine) and in the presence of interferents, the sensor demonstrated high sensitivity, a low detection limit, stability over four weeks, and repeatability of up to 20 consecutive uses, supporting its suitability for practical applications.

Jafari et al. [136] designed a molecularly imprinted electrochemical sensor by electropolymerizing aniline on a graphene oxide and gold nanourchin-modified GCE in the presence of AZM as a template (GNU/GO/GCE). Template extraction produced a porous, rough surface with an increased electroactive area and high conductivity due to the presence of Au nanoparticles. Electrochemical impedance spectroscopy confirmed a high electron transfer rate, enabling enhanced sensitivity and a reduced detection limit. The sensor effectively detected trace levels of AZM in human serum by DPV, showing promise for clinical monitoring.

More recently, Sopaj et al. [137] investigated a screen-printed carbon ink electrode (SPCIE) modified with TiO_2_ nanoparticles for AZM quantification. The incorporation of TiO_2_ increased current response compared with unmodified SPCIE, an improvement attributed to greater surface area, higher roughness, and the electrocatalytic activity of TiO_2_ toward AZM. The modified electrode demonstrated good sensitivity in tap water and human urine without sample pretreatment and selectivity against β-lactam antibiotics such as amoxicillin and penicillin. However, the authors noted that the detection limit and reproducibility require further optimization for reliable field applications. The key analytical parameters and detection performances for specific antibiotic classes are listed in Table 6.

## 5. Perspectives

The global need for reliable detection, quantification, and quality control of widely used drugs continues to drive the development of advanced materials and analytical methods capable of delivering fast, accurate, and cost-effective measurements. Significant progress has been achieved in electrochemical sensor technologies, particularly through the integration of carbon-based materials (CPE, GCE, MWCNT, EPPGE, SPGE, GO, CB, and BDDE) with polymers, metallic nanoparticles, oxides, zeolites, and biorecognition elements for the voltametric and amperometric detection of nonsteroidal anti-inflammatory drugs (NSAIDs) and antibiotics such as naproxen, ibuprofen, aspirin, diclofenac, amoxicillin, cephalexin, and azithromycin. Although the combination of these materials often improves sensor sensitivity and selectivity, it remains unclear whether this is due to genuine synergistic interactions or simply the result of increased electroactive surface area. Addressing this knowledge gap—particularly through detailed studies of electrode–electrolyte interfacial mechanisms—will be critical for the rational design of next-generation sensors.

Although the pursuit of ever lower detection limits (nM to pM) is a common trend in electrochemical sensor research, it is important to recognize that the required sensitivity is application-dependent. For routine quality control of pharmaceutical formulations such as tablets, capsules, and syrups, drug concentrations are typically in the millimolar range, and micromolar sensitivity is sufficient. In these cases, reproducibility, stability, cost-effectiveness, and ease of use are often more critical than achieving ultratrace detection. By contrast, trace-level sensitivity becomes essential in biological fluids (blood, plasma, urine, and cerebrospinal fluid), where drug residues after metabolism and clearance may occur at nM–µM levels relevant for therapeutic monitoring and pharmacokinetics; in environmental samples (surface water, wastewater, drinking water, and soil), where NSAID and antibiotic residues are frequently found at ng/L–µg/L concentrations; and in food safety contexts (milk, meat, and fish), where antibiotic residues may persist at very low concentrations and regulatory limits are often set in the low µg/L range. Therefore, while nanomolar and picomolar detection limits showcase material and design advances, future sensor development should align sensitivity targets with intended applications to ensure practical, clinically meaningful, and environmentally relevant outcomes.

The continuous evolution of electrochemical sensors for the detection of anti-inflammatory and antibiotic drugs reveals a promising trajectory toward more efficient, accessible, and field-deployable analytical tools. Future research should prioritize deeper mechanistic studies at the electrode electrolyte interface to discern true synergistic effects from mere increases in electroactive surface area. This knowledge will be instrumental in guiding the rational design of sensor platforms with optimized sensitivity, selectivity, and reproducibility. Integration of advanced nanomaterials such as doped graphene, hierarchical carbon structures, conductive polymers, and multifunctional nanocomposites offers opportunities to further enhance performance while maintaining cost-effectiveness. Additionally, embedding sustainability and green chemistry principles through eco-friendly electrode materials and scalable fabrication methods will be essential for the long-term adoption of these technologies. Expanding detection capabilities to multiplex analysis and incorporating sensors into miniaturized, wireless, and smartphone-interfaced point-of-care devices could further bridge the gap between laboratory-based assays and real-time, on-site monitoring in clinical, pharmaceutical, and environmental settings. By addressing these directions, electrochemical sensors can evolve into robust, portable, and intelligent platforms capable of safeguarding human and environmental health while meeting the growing demand for rapid and reliable drug detection.

## 6. Conclusions

Electrochemical sensors represent a powerful class of analytical tools for detecting anti-inflammatory and antibiotic drugs, offering high sensitivity, selectivity, rapid response, and cost-effectiveness compared to conventional techniques. The importance of these sensors stems from the continued global relevance of the target molecules: NSAIDs remain among the most consumed drugs worldwide, while antibiotics are essential for infection control but are also central to the urgent challenge of antimicrobial resistance. Both classes of compounds frequently occur as pharmaceutical residues in biological fluids, environmental waters, and food products, raising concerns for human and ecosystem health.

The relevance of advanced electrochemical sensors lies not only in pharmaceutical quality control but also in addressing these broader biomedical and environmental challenges. Their portability, potential for integration into point-of-care and on-site monitoring systems, and adaptability to multiplexed and miniaturized formats make them attractive alternatives for rapid, real-world applications. By enabling reliable detection of trace residues across diverse matrices, these sensors contribute directly to clinical diagnostics, pharmacokinetics, therapeutic monitoring, pollution surveillance, and food safety.

Looking ahead, the applicability of electrochemical sensors will depend on aligning detection capabilities with intended uses—micromolar sensitivity is sufficient for pharmaceutical formulations, while nanomolar and picomolar limits are essential in biological and environmental contexts. Future efforts should prioritize stability, reproducibility, eco-friendly design, and cost-effective scalability to ensure that these platforms evolve into robust, sustainable technologies capable of safeguarding both public health and the environment.

## Figures and Tables

**Table 1:** Summary of Common Electrochemical Techniques for Drug Detection.

Technique	Electrode Configuration	Cell Setup	Analyte Type	Advantages	Reference
Cyclic Voltammetry (CV)	GCE, CPE, BDDE, SPCE	3-electrode system	NSAIDs, antibiotics	Redox mechanism insights, surface studies	[12,13]
Differential Pulse Voltammetry (DPV)	GCE, SPCE, MIP-modified electrodes	3-electrode system	Ibuprofen, aspirin, diclofenac	High sensitivity, low background current	[14,15,16]
Square Wave Voltammetry (SWV)	GCE, CNT-modified, QD based	3-electrode system	Naproxen, azithromycin	Fast scanning, excellent sensitivity	[17,18]
Amperometry (CA/MPA)	Modified SPEs, enzyme based	2- or 3-electrode system	Real-time detection of NSAIDs	Real-time monitoring, simple instrumentation	[19,20]
Electrochemical Impedance Spectroscopy (EIS)	Au, MIP-functionalized, SPCE	3-electrode system	Label-free antibiotic sensors	Interface characterization, high specificity	[21,22]

**Table 2:** Comparison of recognition elements used in electrochemical sensors.

Recognition Element	Advantages	Limitations	Typical Applications
Aptamers	High specificity and affinity; easily synthesized and modified; good stability and reusability	Costly selection process; sometimes reduced performance in complex matrices	Detection of drugs in serum, plasma, and other biological fluids [32,33].
Molecularly Imprinted Polymers (MIPs)	Robust, low-cost, chemically stable; resistant to harsh conditions; easy to prepare	Template removal may be incomplete; nonspecific binding possible	Environmental monitoring (wastewater, surface water), pharmaceutical formulations [34,35].
Enzymes	High specificity; catalytic signal amplification; well established in biosensors	Limited stability; sensitive to pH and temperature; short shelf life	Biosensing of drugs and metabolites, therapeutic monitoring [36,37].
Antibodies	Strong antigen–antibody selectivity; clinically validated	Expensive; sensitive to storage conditions; limited reusability	Immunosensors for pharmaceuticals, diagnostics, and food safety [2,38].
Nanomaterials (pseudo-recognition)	Large surface area; enhance adsorption and electron transfer; tunable catalytic activity	Lack intrinsic molecular specificity; potential reproducibility issues	General sensing, particularly for nonbiological matrices and trace-level detection [39,40].

**Table 3:** Electrochemical sensors for anti-inflammatory drugs.

Drug	Electrode Material	Technique	LOD (mM)	Reference
Ibuprofen	GCE/aptamer to gold-nanoparticles	DPV	0.0000005	[48]
Aspirin	GCE/GO	SWV	0.021	[56]
Naproxen	ZnO–MWCNT	SWV	0.010	[57]
Diclofenac	GCE/Pt nanoflowers + rGO	DPV, CV	0.003	[15]
Ketoprofen	GCE/MIP-ZrMOFs/RGO	DPV	0.00022	[58]
Indomethacin	CPE/Gold NPs	SWV	0.00068	[59]
Piroxicam	Fe_3_O_4_-GO-PCA	DPV	0.056	[60]
Meloxicam	GR/GCE	SWV	0.066	[61]
Celecoxib	CPE/Ag-NPs-Ch-GO	CV	0.00251	[62]
Etodolac	Poly vinyl chloride	DPV	4.66	[19]

GCE—glassy carbon electrode; CPE—carbon paste electrode; SPCE—screen-printed carbon electrode; GO—graphene oxide; rGO—reduced graphene oxide; AuNPs—gold nanoparticles; MWCNT—multiwalled carbon nanotubes; Pt nanoflowers—platinum nanostructures with flower-like morphology; MIP—molecularly imprinted polymer; ZrMOFs—zirconium-based metal–organic frameworks; Fe_3_O_4_—magnetite (iron oxide); PCA—p-chloranilic acid; GR—graphene; Ag-NPs—silver nanoparticles; Ch—chitosan.

**Table 6:** Electrochemical Sensors for Azithromycin.

Electrode/Modification	Technique(s)	Linear Range	LOD	Matrix/Application	Notes	Reference
MWCNTs nanocomposite decorated with MgCr_2_O_4_ spinel	DPV	0.25–4.0 and 4.0–10.0 μm	0.07 µM	Pharmaceutical, plasma, urine	Hydrophobic and electrostatic interaction enhanced adsorption	[134]
Aniline-MIP/GCE	Not specified	0.3–920.0 nM	0.1 nM	Pharmaceutical	High electron transfer rate	[136]
EGr/GCE	Amperometry	0.01–10 µM	3.03 nM	Pharmaceutical drugs	High selectivity and reusability	[139]
Fumed silica/CPE	DPV	44.0–1000.0 µM	11 mM	Pharmaceutical, plasma, urine	Sensor showed high sensitivity and stability	[142]
Poly-threonine carbon paste electrode (PTCPE)	SWV	8.88–1000.0 µM	0.32 µM	Pharmaceutical formulations	High sensitivity and stability	[143]
Gr/IL/GCE	DPV	0.65–38 µM	0.25 µM	Pharmaceutical formulations	Enhanced electron transfer	[144]
Molecularly imprinted polymer (MIP)	CV, EIS	13.33 nM–66.67 μM	0.85 nM	Biological fluids	Rapid mass transfer and high surface area for binding	[145]
Graphene nanoribbon + ionic liquid/CPE	SWV	10 µM–2 mM	0.66 µM	Pharmaceutical and Biological Samples	Ionic liquid enhances conductivity	[146]
Molybdenum disulfide/titanium aluminum carbide/ GCE	LSV	0.05–25 µM	0.009 µM	Pharmaceutical and Biological Samples	2D nanocomposite improves electron transfer and structural stability	[147]
MIP/acetylene black/CPE	DPV	0.2–20 µM	2.3 µM	Pharmaceutical and Biological Samples	Superior current response and high selectivity	[148]
